# Ethereal Tincture of Valerian in Convulsions of Children

**Published:** 1860-10

**Authors:** H. L. Byrd

**Affiliations:** Savannah, Ga.


### ARTICLE V.

[For the Atlanta Medical and Surgical Journal.]

"*Ethereal Tincture of Valerian in Convulsions of Children.*"

Mr. Editor: The above is the caption of an article published in the original department of your Journal for Sept., purporting to have been written by Dr. B. W. Hardee, of Savannah. Said article requires a few words of notice from me, not, however, in any spirit of anger, but ot regret. I regret that he should have pursued the course which he has done, for several reasons, and among them, that he should have felt willing to make statements in regard to the use of the "Ethereal Tincture of Valerian in the Convulsions of Children," without consultation with myself before doing so, or, at all events, indicating the *source* from which *his* knowledge was derived; especially, when he was perfectly familiar with *all* the circumstances bearing upon the origin of the treatment in question.

I have been using Ethereal Tincture of Valerian—or Volatile Tincture of Valerian, as it is sometimes called—in the several forms of eclampsia, for the last twenty years, and in the convulsions of childhood, it has occupied a prominent place in my *armamentarium therapeia* for the management of such cases. I contemplate publishing, hereafter, my *own* experience with this, and other preparations of Valerian, in the treatment of certain morbid conditions of the system. Dr. Hardee was either ignorant of the fact that the Ethereal Tincture, to which he alludes, is *not* officinal, or he forgot to mention the formula for its preparation. I deem it a matter of sufficient importance to your readers to state the formula, in order that the article may be properly understood and appreciated. I have had it prepared for use by three or four Druggists in Savannah, according to the following:

℞—Rad. Valerian, centers, ℥ ij.,

Sulph. Ether, ℈j.

Macerate for ten or twelve days, when it is ready for use. Dr. Hardee obtained his first supply of the preparation from one of the establishments to whom I had furnished the recipe, as I told him what the article was, and where it could be procured. I arrogate to myself no claim to the preparation as a discoverer. I furnished the formula to the dispensing apothecaries alluded to, with a view to the general introduction of the preparation into the practice of our physicians.

I trust that Dr. Hardee's overweening "desire merely to call the attention of the profession to the use, &c.," of remedial agents, will not urge him again to forestall his medical brethren in the publication of such information as they may desire to "dress up" according to their *own tastes*, and present them in their *own way* to the consideration of the Faculty. Very Respectfully, Yours,

Savannah, Sept. 22, '60. H. L. BYRD, M. D.

