# Errata

**Published:** 1860-11

**Authors:** 


### Errata.

We regret amongst others, the typographical errors which occurred in the formula for the preparation of "Ethereal Tincture of Valerean," in the communication of Dr. H. L. Byrd, of Savannah, in our last issue. The formula should read:

℞.—Rad. Valerian, contused, ℥ ii.

Sulph. Ether, Oi.

Macerate for ten or twelve days, when it is ready for use.

